# Dual activity of PD-L1 targeted Doxorubicin immunoliposomes promoted an enhanced efficacy of the antitumor immune response in melanoma murine model

**DOI:** 10.1186/s12951-021-00846-z

**Published:** 2021-04-13

**Authors:** María Merino, Teresa Lozano, Noelia Casares, Hugo Lana, Iñaki F. Troconiz, Timo L. M. ten Hagen, Grazyna Kochan, Pedro Berraondo, Sara Zalba, María J. Garrido

**Affiliations:** 1 Department of Pharmaceutical Technology and Chemistry, School of Pharmacy, University of Navarra, 31008 Pamplona, Navarra Spain; 2 Program of Immunology and Immunotherapy, CIMA-Universidad de Navarra, Pamplona, Spain; 3 Navarra Institute for Health Research (IdisNA), Pamplona, Spain; 4 Laboratory of Experimental Oncology, Erasmus Medical Center, Rotterdam, The Netherlands; 5 Department of Oncology, Navarrabiomed-Biomedical Research Centre, Pamplona, Spain

**Keywords:** Liposome, PD-L1, Doxorubicin, Chemo-immunotherapy, Immunotherapy, Immunogenic cell death, Melanoma, Immunocheckpoint

## Abstract

**Background:**

The immunomodulation of the antitumor response driven by immunocheckpoint inhibitors (ICIs) such as PD-L1 (Programmed Death Ligand-1) monoclonal antibody (α-PD-L1) have shown relevant clinical outcomes in a subset of patients. This fact has led to the search for rational combinations with other therapeutic agents such as Doxorubicin (Dox), which cytotoxicity involves an immune activation that may enhance ICI response. Therefore, this study aims to evaluate the combination of chemotherapy and ICI by developing Dox Immunoliposomes functionalized with monovalent-variable fragments (Fab’) of α-PD-L1.

**Results:**

Immunoliposomes were assayed in vitro and in vivo in a B16 OVA melanoma murine cell line over-expressing PD-L1. Here, immune system activation in tumor, spleen and lymph nodes, together with the antitumor efficacy were evaluated. Results showed that immunoliposomes bound specifically to PD-L1^+^ cells, yielding higher cell interaction and Dox internalization, and decreasing up to 30-fold the IC_50_, compared to conventional liposomes. This mechanism supported a higher in vivo response. Indeed, immunoliposomes promoted full tumor regression in 20% of mice and increased in 1 month the survival rate. This formulation was the only treatment able to induce significant (p < 0.01) increase of activated tumor specific cytotoxic T lymphocytes at the tumor site.

**Conclusion:**

PD-L1 targeted liposomes encapsulating Dox have proved to be a rational combination able to enhance the modulation of the immune system by blocking PD-L1 and selectively internalizing Dox, thus successfully providing a dual activity offered by both, chemo and immune therapeutic strategies.

**Graphic Abstract:**

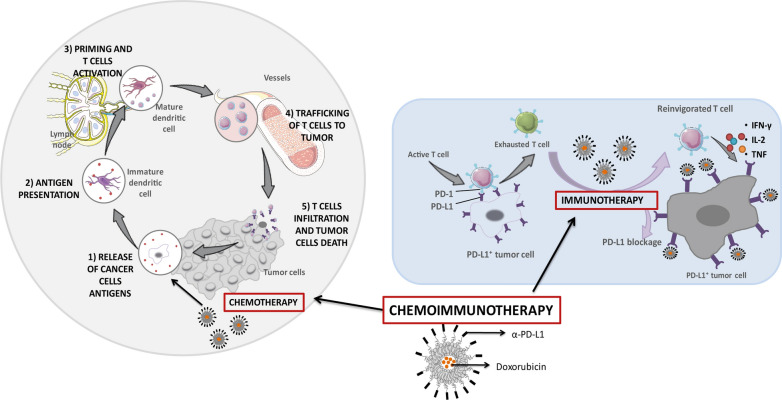

**Supplementary Information:**

The online version contains supplementary material available at 10.1186/s12951-021-00846-z.

## Background

The immune system is able to detect and eliminate the majority of tumors at early stages of development [1]. However, cancer cells can evade this process by modulating certain mechanisms such as the up-regulation of immune checkpoints.

Programmed Death-ligand-1 (PD-L1) [2–5] is an immune checkpoint commonly over-expressed in tumor cells that binds to the Programmed Death-1 (PD-1) receptor, present on activated effector T cells [3, 6, 7]. At the tumor microenvironment, the interaction of PD-1/PD-L1 induces the down-regulation of T cell antitumor activity and the secretion of inhibitory cytokines, causing immunosuppression and promoting tumor progression [3, 7].

In recent years, blockade of the PD-1/PD-L1 axis using specific monoclonal antibodies has become a promising approach in oncology [2–5]. These immune checkpoint inhibitors (ICIs) have shown relevant clinical outcomes [2, 3, 8–12]. However, the high inter-individual variability observed with these treatments has led to the search for rational combinations with other strategies such as chemotherapeutic agents, to increase the benefit of the immunotherapy [7, 13, 14].

In this context, Doxorubicin (Dox) is a good candidate for combination with ICIs. Dox induces cytotoxicity, releasing tumor antigens, which may contribute efficiently to stimulate the immune system by the immunogenic tumor cell death mechanism that participates promoting the infiltration of effector CD8^+^ T cells, responsible for the antitumor effect [15–18].

Currently, Dox is administered into liposomal formulations to reduce certain life-threatening side effects, in particular cardiotoxicity [18–20]. Furthermore, liposomes tend to passively accumulate in tumor tissue by the Enhanced Permeability and Retention effect, a mechanism driven by the rapid tumor growth that generates immature leaky new blood vessels that allows an increased exposure to the drug in the tumor site [21–23].

On the other hand, immunoliposomes or liposomes decorated at the surface with whole monoclonal antibodies or variable monovalent fragments (Fab’), promote active targeting [24] by a selective binding to the target, which must be upregulated in cancer cells. This mechanism increases intracellular drug bioavailability [25] and thereby, improves antitumor drug efficacy [24–26].

Accordingly, PD-L1, commonly over-expressed in many solid cancers and often involved in CD8^+^ T cell exhaustion, represents an attractive target for immunoliposomes [27–29]. In fact, our group has developed empty PD-L1 targeted liposomes that have demonstrated selective binding and a timid immune modulation of effector T cells in a melanoma murine model [30]. However, in this study the aim is to evaluate the advantages derived from the combination of chemo-and immunotherapy developing Dox-liposomes functionalized with Fab’ of α-PD-L1. Our hypothesis states that immunoliposomes may induce the reversion of the immunosuppressive tumor microenvironment by blocking the PD-1/PD-L1 interaction and contributing to increase cell internalization of Dox and thereby, drug cytotoxicity and immunogenic cell death activity, a dual mechanism able to promote an enhancement of the antitumor response.

## Results

### Immunoliposomes yielded a reproducible and stable nanoplatform

The film-hydration method provided homogenous liposomal populations, as is listed in Table 1. Indeed, the three formulations, conventional empty liposomes (LP), conventional or non-targeted Dox liposomes (LPD) and Fab’-anti-PD-L1 Dox liposomes or targeted liposomes (LPF) (Additional file 1: Table S1), showed similar physicochemical characteristics, demonstrating that the encapsulation of Dox or the conjugation of Fab’ did not influence the parameters.

**Table 1:** Physicochemical characterization of the different liposomal formulations. Data represent the mean ± SD of three independent batches

	LP	LPD	LPF
Particle size (nm)	115.1 ± 1.80	116.7 ± 1.10	121.5 ± 0.80
PDI	0.097 ± 0.020	0.052 ± 0.013	0.082 ± 0.023
Zeta potential (mV)	− 27.6 ± 3.48	− 23.5 ± 1.87	− 23.9 ± 0.72

PDI: Polydispersity index; LP: conventional empty liposomes; LPD: conventional Dox liposomes; LPF: targeted Dox liposomes

The efficiency of encapsulation was ≥ 90 ± 3.8% in all cases, while the efficiency of antibody conjugation was 37.9 ± 6.02%.

**Fig. 1:**
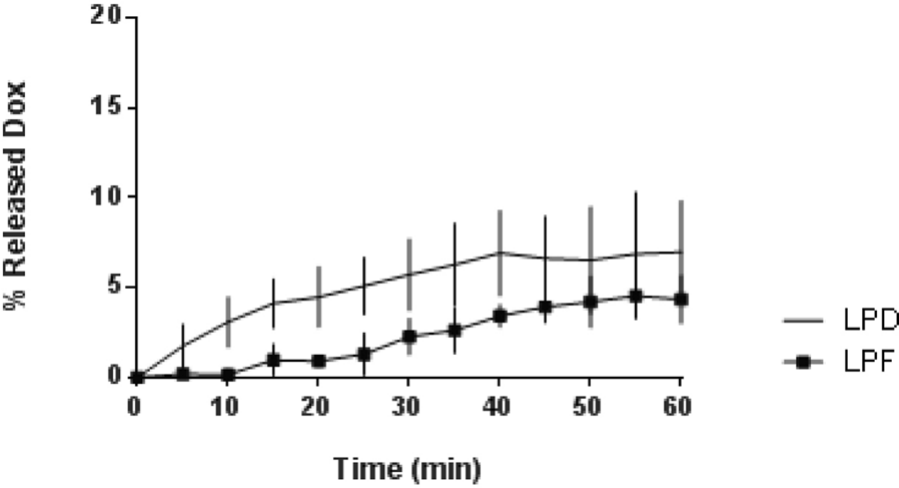
Dox release profile of Dox liposomes and Dox immunoliposomes in FBS. Each point represents mean ± SD of three different batches analysed in duplicate. No statistical differences were found. Dox: Doxorubicin; LPD: conventional Dox liposomes; LPF: targeted Dox liposomes; FBS: fetal bovine serum

Accumulative drug release curves in Fig. 1 show that Dox released during 1 h from both formulations, LPD and LPF, was very low (< 10%), supporting an adequate stability for in vivo administration. In addition, the long-term stability of formulations in Hepes saline (pH 6.7) at 4 °C was evaluated for 3 months. Results for particle size, EE (%), PDI or surface charge did not change (data not shown).

### Immunoliposomes enhanced cellular uptake and cytotoxic effect

**Fig. 2:**
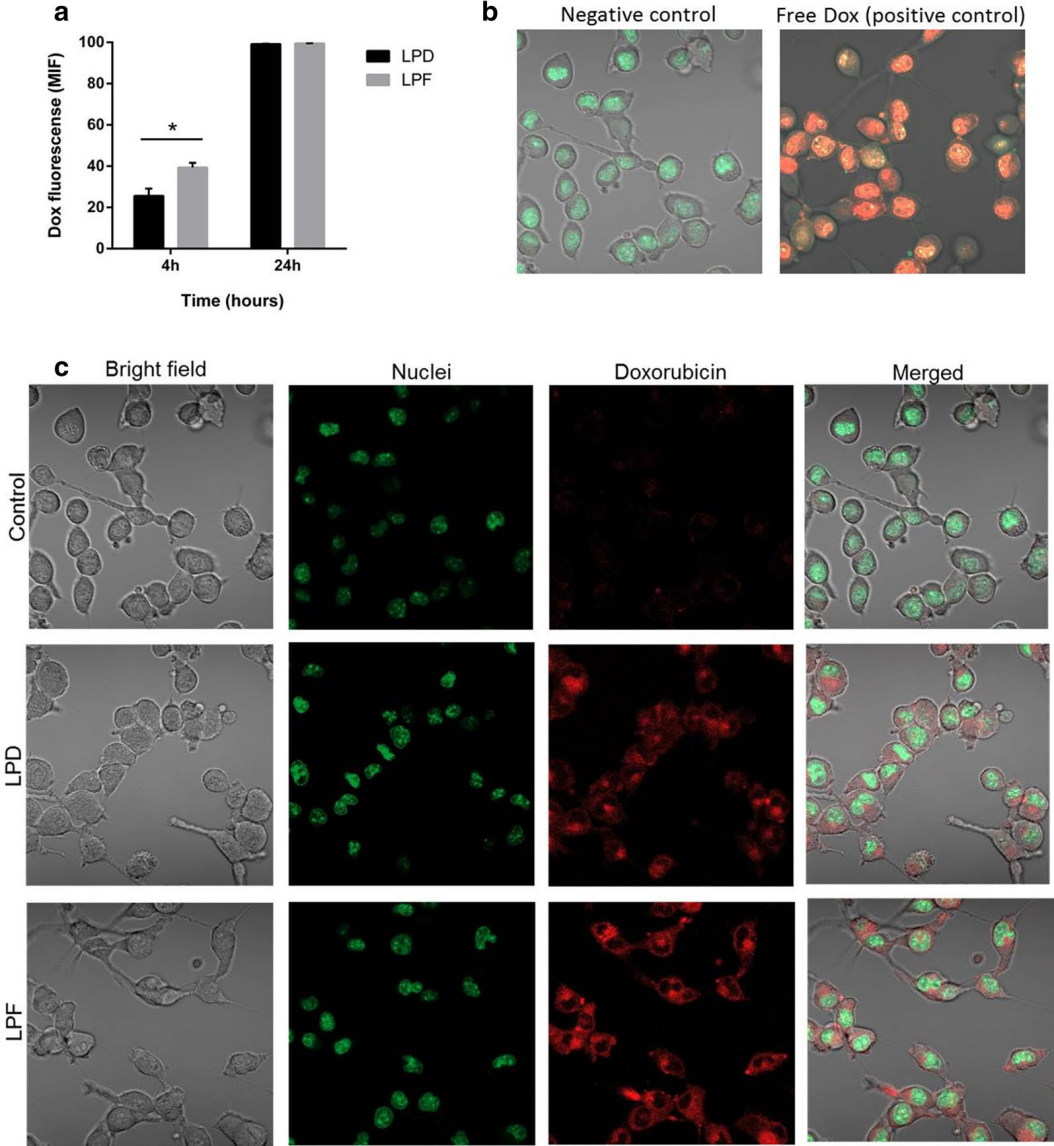
Doxorubicin fluorescent signal in B16OVA cells exposed to liposomes. **a** Fluorescent signal measured by flow cytometry after 4 and 24 h incubation with LPD and LPF. Bars represent the mean ± SD of three independent studies. **b** Confocal microscopy images of positive (free Dox treatment) and negative (untreated) controls; **c** Cellular images after 4 h exposure to LPD and LPF. The fluorescent signals: red for Dox and green for the nuclei. Dox: doxorubicin; LPD: conventional Dox liposomes; LPF: targeted Dox liposomes; MIF: mean intensity fluorescence signal

Cell uptake for targeted and non-targeted liposomes encapsulating Dox is represented in Fig. 2 (panel a). The intracellular mean fluorescent signal (MIF) measured after 4 h exposure was statistically higher for targeted than for non-targeted liposomes, confirming the selectivity of the ligand by the PD-L1 expressed in B16OVA cells. This finding, corroborated by confocal microscopy in Fig. 2c, shows that although both formulations reach the cytoplasm, the targeting provided higher signal intensity. However, the non-differences found after 24 h of continuous exposure suggest that the impact of the targeting is particularly relevant at short exposure times, whereas for longer times, non-targeted liposomes might achieve an equilibrium across the membrane. The free drug, with rapid access to the nucleus, was used to illustrate the positive control (Fig. 2b).

### Immunoliposomes induced higher cytotoxicity

**Fig. 3:**
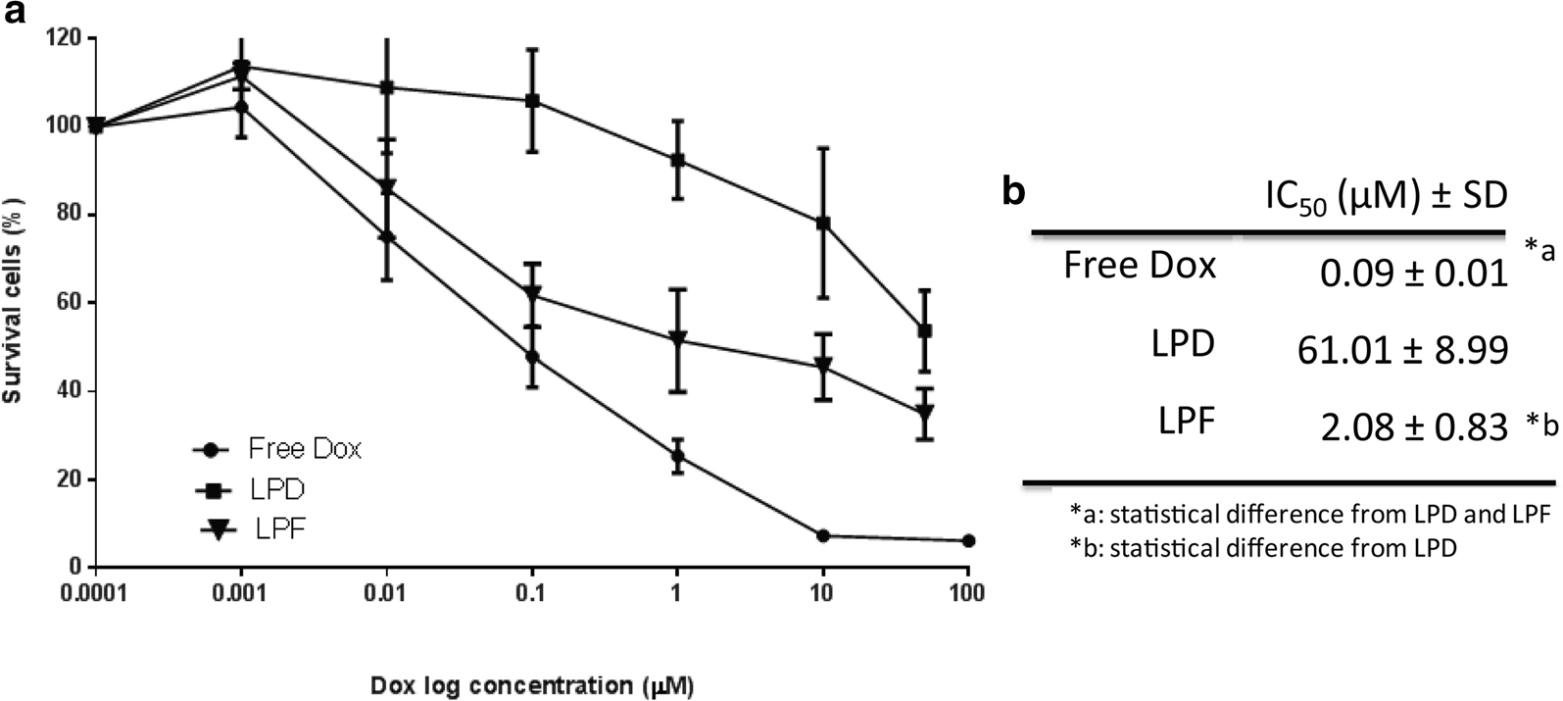
Cytotoxic activity of free and encapsulated Dox at 72 h after a 4 h exposure to treatments. **a** Survival curves (or cell viability curves) of B16OVA cells exposed to increasing Dox concentrations; **b** IC_50_ concentration values. Data represent the average ± SD of three independent studies. Dox: doxorubicin; LPD: conventional Dox liposomes; LPF: targeted Dox liposomes; IC_50_: Dox concentration able to inhibit 50% of cell proliferation compared to control

Cytotoxicity exerted by Dox, both free and encapsulated, after 4 h of exposure was assayed 72 h later. Figure 3b summarizes the IC_50_ values, observing that the free drug, or positive control, displayed the highest cytotoxic activity, followed by the targeted liposomes with 30 times higher potency than LPD. This low activity of LPD is probably associated with the unspecific cell uptake (Fig. 2a) and the slow drug release rate.

Interestingly, the free Dox and LPF displayed similar cell viability profiles at low concentrations (< 1 µM), suggesting that the receptor binding, internalization and Dox release from the formulation rendered a drug bioavailability similar to the free drug (Fig. 3a).

### Immunoliposomes were cleared faster than non-targeted liposomes

**Fig. 4:**
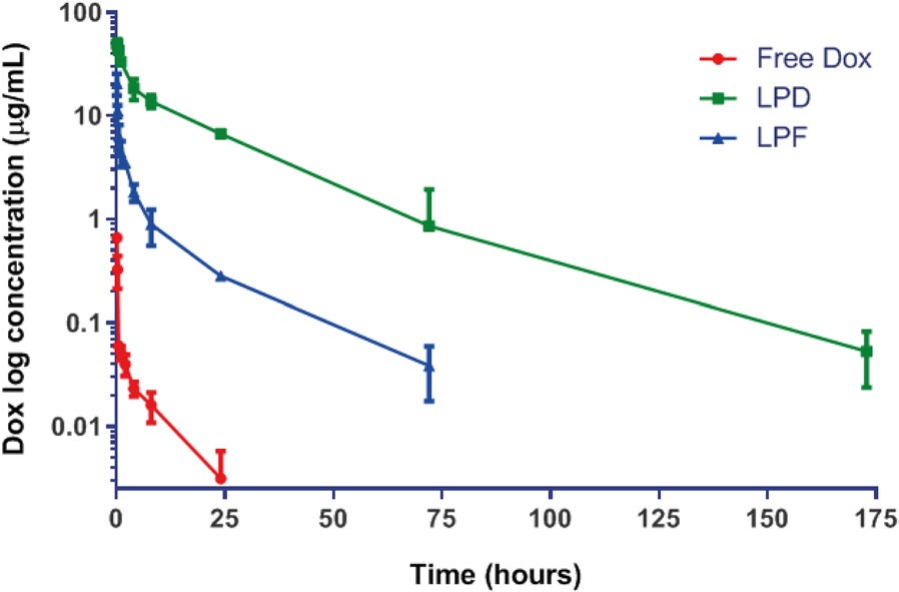
Pharmacokinetics evaluation. The average of plasma concentrations (n = 3 mice) per time point is represented by symbols together with the mean standard deviation. Lines correspond to the interpolation data. Data were collected from B16OVA tumor bearing mice treated with a single intravenous dose (3 mg/kg) of free Dox, Conventional Dox liposomes (LDP) and Targeted Dox liposomes or immunoliposomes (LPF)

Time profiles of Dox plasma concentrations measured in melanoma-bearing mice treated with free and encapsulated drug are represented in Fig. 4. Dox was quantified by HPLC (Additional file 1: Figure S1).

Table 2 lists the pharmacokinetic parameters calculated by non-compartmental analysis. Differences in the t_1/2_ between non-targeted (21 h), and targeted liposomes, (11 h) were also reflected in the drug exposure expressed as AUC_0-∞_. In line with these differences, total plasma clearance was approximately 15 times higher for LPF compared to conventional pegylated liposomes. In contrast, free Dox, widely distributed and rapidly eliminated, achieved the lowest exposure.

**Table 2:** Dox pharmacokinetic parameters calculated by a non-compartmental analysis after 3 mg/kg dose of free Dox, LPD and LPF intravenously administered to tumor bearing mice

	t_1/2_ (h)	AUC_0-∞_ (µg h/mL)	Cl (mL/h)	Vd (mL)
Free Dox	6.99	0.60	99.6	1005.47
LPD	21.83	586.58	0.1	3.15
LPF	11.31	37.96	1.6	25.88

t_1/2_: Elimination half life; AUC: area under the curve of Dox plasma concentrations vs. time; Cl: total plasma clearance; Vd: volume of distribution; LPD: conventional Dox liposomes; LPF: targeted Dox liposomes; Dox: doxorubicin

### Immunoliposomes triggered local and systemic antitumor immune response

The immune response exerted by the different formulations was characterized in B16OVA tumor-bearing mice injected with a single dose, 3 mg/kg, of free Dox, LPD, LPD/free α-PD-L1 (28 µg/mouse) and LPF.

**Fig. 5:**
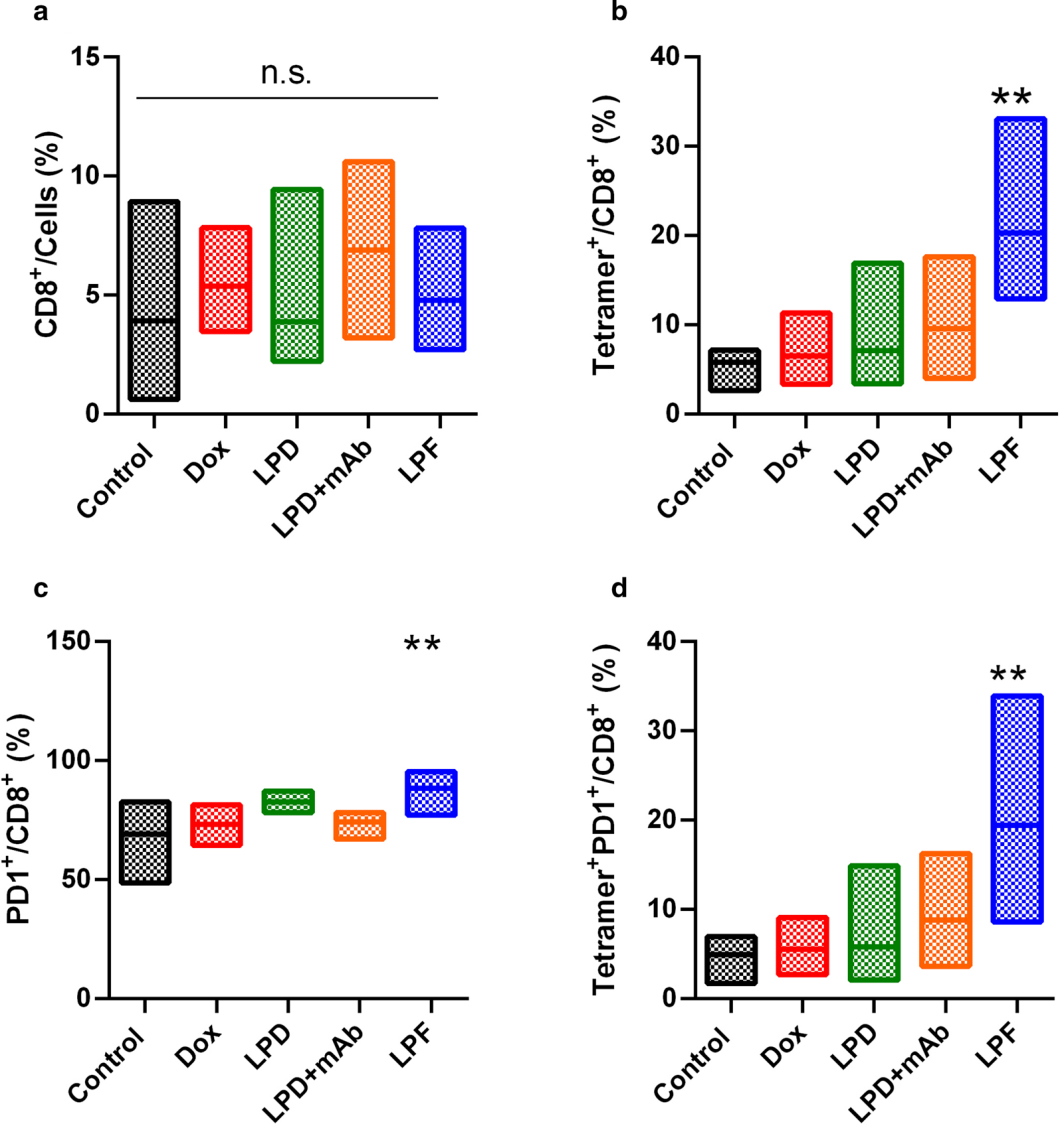
Immune response promoted by different treatments in tumor tissue. B16OVA tumor bearing mice were administered with different treatments: saline, free Dox, Conventional Dox liposomes (LPD), LPD co-administered with 28 µg of free α-PD-L1 (mAb) or Targeted Dox liposome or immunoliposomes (LPF). Dox was intravenously injected at 3 mg/kg and eight days later, tumors were removed to evaluate different T cell subpopulations. **a** CD8^+^ cells; **b** Tumor specific cytotoxic T lymphocytes (Tetramer^+^/CD8^+^); **c** Activated cytotoxic T lymphocytes (PD1^+^/CD8^+^); **d** Activated CD8^+^ tumor specific lymphocytes. Bars show the minimum and maximum value with the mean of each treatment. Data correspond to two independent assays. n = 8 in total **p < 0.01 compared to control group

In tumor, CD8^+^ T cell levels were not statistically different across treatments (Fig. 5a); however, targeted liposomes promoted a significant increment of specific and active tumor infiltrating T cells (TILs) (Fig. 5b–d). Indeed, in a deeper analysis of these TILs, mice treated with LPF presented a significant (p < 0.01) increase in tumor specific T cells (Tetramer^+^/CD8^+^) and activated tumor specific lymphocytes (PD1^+^Tetramer^+^/CD8^+^), reflecting a specific antigen response at the local target. This response was not induced by other treatments, including the combination of non-targeted liposomes and free α-PD-L1.

**Fig. 6:**
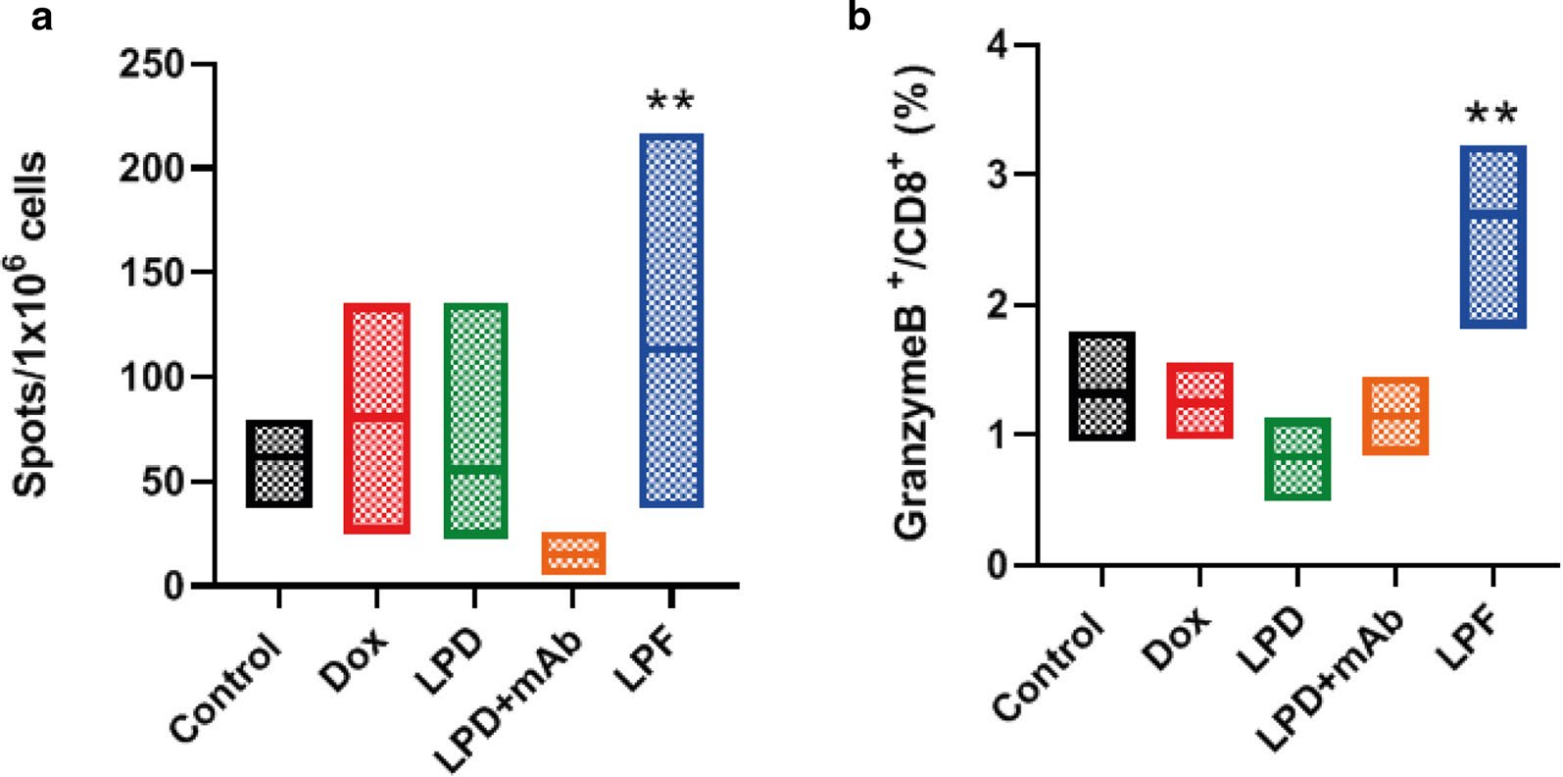
Systemic immune response was measured in spleen and lymph nodes collected from B16OVA tumor bearing mice treated with saline, free Dox, Conventional Dox liposomes (LPD), LPD co-administered with 28 µg of free α-PD-L1 (mAb) or Targeted Dox liposome or immunoliposomes (LPF). Dox was intravenously injected at 3 mg/kg and eight days later, organs were removed to analyze different T cell subpopulations. **a** Lymphocyte activation status determination in spleen after incubation with SIINFEKL peptides (ELISPOT); **b** Degree of activation (Granzyme B) measured in CD8^+^ cells in lymph nodes after 4 h of stimulation with PMA/Ionomicin. Bars show the maximum and minimum range of data with the mean. Data belong to two independent studies involving eight mice. **p < 0.01 compared to control

Interestingly, this immune status in tumor was in line with the systemic immune response analyzed in the spleen and lymph nodes, which was also higher after immunoliposomes administration (Fig. 6a). Note that in the spleen, after the SIINFEKL pulse, LPF induced a strong CD8^+^ activation; whereas in the lymph nodes, an increase in Granzyme B expression in CD8^+^ cells was found, as is represented in Fig. 6b. Therefore, immunoliposomes exerted a systemic efficient activation of immune response, strengthening the tumor response, whereas this effect was not outperformed either by the chemotherapy or non-targeted liposomes alone or by the combination of LPD and free α-PD-L1.

### Immunoliposomes were able to efficiently control the tumor growth

B16OVA melanoma-bearing mice were treated with 3 mg/kg of Dox, free and encapsulated on LPD, LPF and LPD/α-PD-L1 (28 µg/mouse), every 72 h during 3 cycles.

**Fig. 7:**
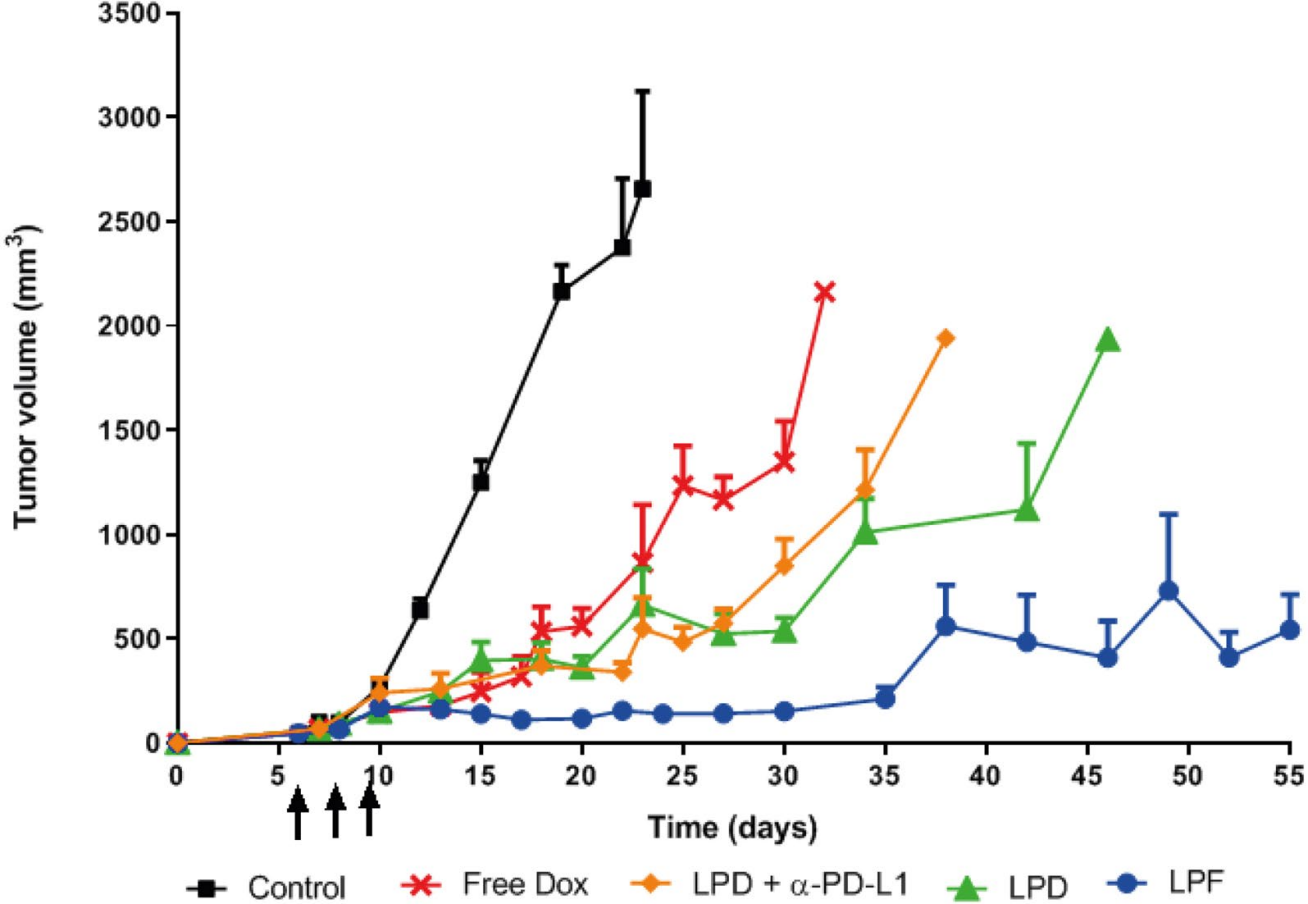
Profiles of tumor growth measured in B16OVA tumor bearing mice intravenously administered with three cycles (cycle/every 72 h) of different treatments: saline, free Dox, Conventional Dox liposomes (LPD), LPD co-administered with 28 µg of free α-PD-L1 or Targeted Dox liposome or immunoliposomes (LPF). Dox was intravenously administered at 3 mg/kg and tumor growth was measured twice a week. Lines represent the average and standard deviation of 6 mice per group of treatment and arrows the three administrations

All treatments provided an antitumor effect compared to the control group, as depicted in Fig. 7. However, encapsulated Dox was associated with more effective tumor shrinkage than with the free form. In addition, non-targeted liposomes in monotherapy or combined with free α-PD-L1 behave similarly, demonstrating that the 28 μg/mouse dose of the free antibody did not contribute significantly to the drug effect. In contrast, immunoliposomes achieved an efficient control of tumor growth for more than 40 days, as is observed in Fig. 7.

**Fig. 8:**
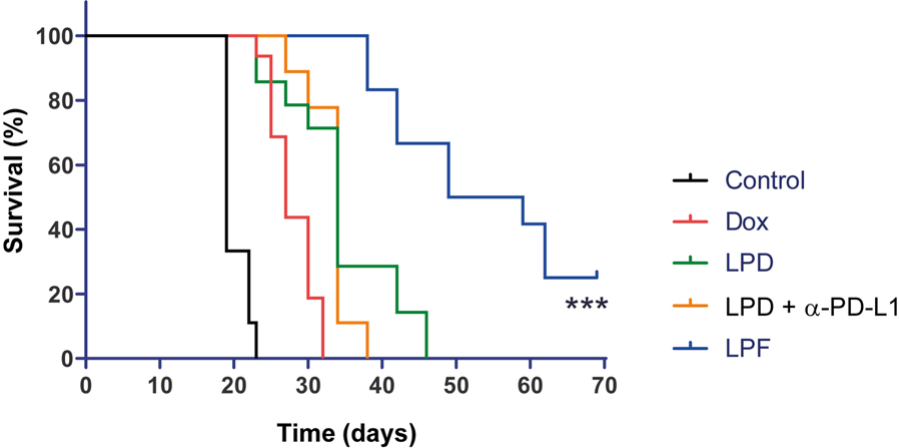
Survival curves for the different treatments assayed in B16OVA melanoma-bearing mice. B16OVA tumor bearing mice intravenously administered with three cycles (cycle/every 72 h) of different treatments: saline, free Dox, Conventional Dox liposomes (LPD), LPD co-administered with 28 µg of free α-PD-L1 or Targeted Dox liposome or immunoliposomes (LPF). Dox was intravenously administered at 3 mg/kg and tumor progression was measured twice per week. Immunoliposomes increased significantly the survival rate in comparison with the other treatments (***p < 0.001)

In fact, the Kaplan-Meyer curve shows that the overall survival for this group was 70 days, which represents a statistically significant (p < 0.0001) increase in the life-span of almost 1 month compared with the non-targeted formulation. Indeed, the efficacy of LPD and Dox was similar (Fig. 8). No significant side effects such as loss of body weight or ulceration were found during the experiments (Additional file 1: Figure S2).

**Fig. 9:**
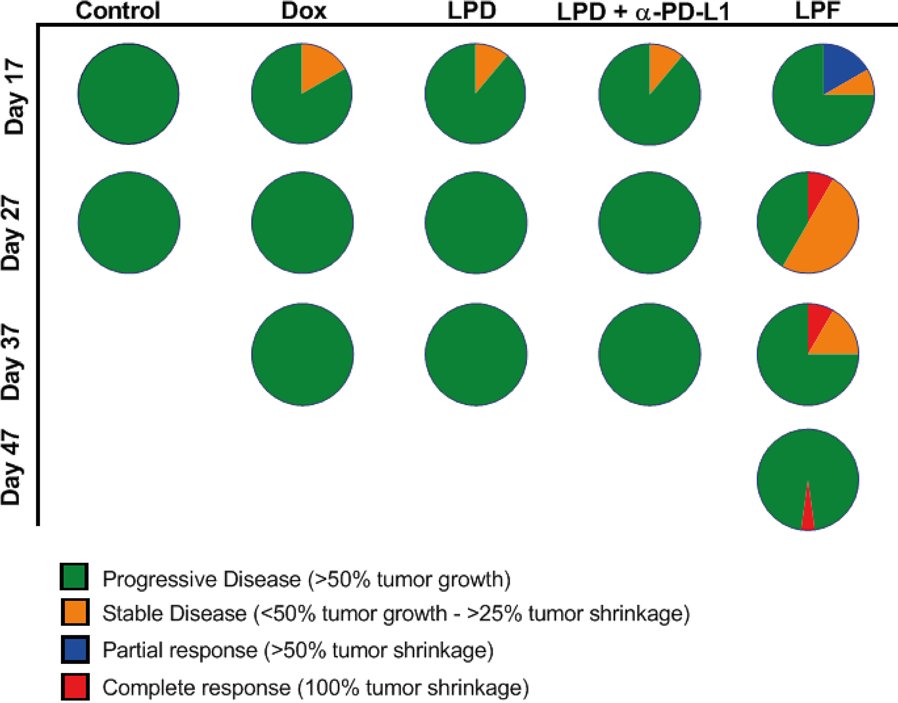
RECIST criteria applied to the therapeutic assay of liposomes. B16OVA tumor bearing mice intravenously administered with three cycles (cycle/every 72 h) of different treatments: saline, free Dox, Conventional Dox liposomes (LPD), LPD co-administered with 28 µg of free α-PD-L1 or Targeted Dox liposome or immunoliposomes (LPF). Dox was intravenously administered at 3 mg/kg and tumor progression was measured twice per week. The values, expressed as percentage, were calculated comparing the tumor size at a specific time point with its initial size at day 7 after cell inoculation

Finally, the impact of the therapies on the antitumor effect was evaluated applying the RECIST (Response Evaluation Criteria In Solid Tumors) criteria [31, 32]. Figure 9 summarizes tumor growth dynamics over time, showing that at the end of treatments (day 17), all treated groups achieved a stable disease in a small percentage of mice, but the effect was transient. However, LPF was the only treatment that attained total tumor regression in 20% mice at day 27, a percentage that remained stable until the end of the experiment (day 69). In addition, 50% of mice treated with LPF presented stable disease at day 27, suggesting that a new cycle of treatment might be beneficial.

## Discussion

Currently, immunotherapy, in particular ICIs, has become a solid pillar for cancer treatment. Clinical benefits such as total and long-term tumor remission in a minority of patients, have led investigators to use different therapeutic combinations to increase that number of patients [14, 33]. In this context, Dox based-combinations with different immune-approaches have been previously reported by several authors as a promising strategy [34–38]. Accordingly, the main objective of the present study was to develop and evaluate a novel single and versatile nano-liposome combining Dox and α-PD-L1. To address this, our group formulated a new PD-L1 targeted nanoplatform to block selectively this immune checkpoint [30]. Nevertheless, in this work, we present a more advanced liposome encapsulating Dox to exploit the advantages of that nanoplatform, demonstrating enhancement not only of the antitumor immune effect, but also efficacy.

Here, the optimization of the methodology previously reported by Merino et al. (2019) [30] led us to formulate highly reproducible liposomes in terms of encapsulation, particle size, PDI or stability, providing similar formulations with independence of the targeting [38–41].

The impact of the selective targeting assayed in B16OVA cells, overexpressing PD-L1 (PD-L1^+^), has resulted in the reduction of the IC_50_ of Dox compared to non-targeted liposomes. This PD-L1 selectivity yielded higher drug uptake and thereby, higher intracellular bioavailability, similar to the result reported for gastric cancer cells overexpressing PD-L1, exposed to nanoparticles conjugated with α-PD-L1 [42]. There, the cellular uptake correlated with a more effective inhibition of PD-L1 expression [42], but non-in vivo data were further explored. However, the in vivo study is crucial to know the therapeutic benefit and pharmacokinetics behaviour. In this work a significant change in drug exposure across targeted, non-targeted and free drug was found. Indeed, the free drug presenting the highest cytotoxicity in the in-vitro studies did not correlated with the highest efficacy in in-vivo.

Although PK parameters calculated using Dox mean concentration values, did not provide information about inter-individual variability or associated bias, results are in line with the literature [38, 40, 41]. Thus, free Dox was widely distributed and rapidly eliminated, a characteristic associated with limited efficacy, as is observed here (Fig. 7) and serious adverse effects, justifying drug encapsulation into liposomes [20, 43–45]. This strategy provided long-circulating liposomes, reducing Dox elimination and therefore, increasing the drug exposure, which was much higher than for immunoliposomes. This different behaviour, commonly reported in the literature for targeted liposomes [38, 43], can be explained by the ligand selective binding, proved in vitro, which promotes faster clearance in comparison with conventional pegylated liposomes [38, 43]. Furthermore, the present targeted formulation might also bind to the PD-L1 expressed in certain blood circulating factors such as exosomes, soluble PD-L1 or immune cells, thus contributing to the targeted liposomes elimination [46–48]. In this regard, our group performed an ex-vivo assay. Differentiated myeloid cells collected from murine bone marrow, were incubated with fluorescent targeted and non-targeted liposomes at different amounts of lipids. Cell uptake was much higher for targeted liposomes, correlating with a dramatic reduction of PD-L1 expression (Figure S3). Altogether, these findings support the involvement of myeloid cells in the capture of targeted liposomes. In this way, Xiong et al. [49] have reported that the PD-L1 targeting to myeloid cells associated with depleting agents might be a good strategy to induce functional remodeling of macrophage compartment, enhancing the efficacy of α-PD-L1 treatment.

However, despite the PK differences, the amount of targeted liposomes in the tumor was sufficient to induce a significant increase in activated tumor-specific cytotoxic T-cells (Fig. 5). Hence, targeted liposomes increased PD1^+^ /CD8^+^ T cells in tumor microenvironment, indicating that the PD-L1 blockage might reinvigorate antitumor immune function, which would be facilitated by Dox cytotoxicity, increasing the antigen release. In this line, Gurung et al. (2020) [50] found a reinvigoration of CD8^+^ T-cell after nine cycles of treatment with specific PDL-1-binding peptides attached to Dox-liposomes in CT26 colon tumor model. This result supports our hypothesis about the contribution of Dox in the ICI activity in a synergistic manner or at least, in a potentiation. In fact, the dose of Dox was the same for all treatments. However, Dox was not able to induce the same response, in particular the free Dox, associated with the lower efficacy. Furthermore, LPD co-administered with α-PD-L1 at the dose corresponding to LPF (28 µg/mouse) did not render any immune response. Interestingly, 28 µg/mouse is far from 100 or 200 µg/mouse, the generally used dose for α-PD-L1 efficacy [8] or empty PD-L1 targeted liposomes [30]. Therefore, Dox contributed to immune response activation only after immunoliposome administration.

Besides, targeted liposomes enable an immune activation in the spleen and lymph nodes that would support the modulation observed in tumor. Thus, the increase in CD8^+^ T cells expressing Granzyme B reflects a specific activation of tumor-infiltrating T cells [8], even when non-differences in CD8^+^ T cell numbers were observed across treatments. Note that TILs activation, expansion and infiltration in the tumor involve a dynamic process, which must be taken into consideration when selecting times for tumor and organ collection (spleen or lymph node), in order to detect the highest difference in TIL levels [8, 51].

To our knowledge, this is the first immunoliposomal formulation that demonstrates systemic and local enhancement of the antitumor immune response, correlating with the efficacy. Importantly, a remarkable delay in tumor growth and tumor shrinkage was observed after immunoliposomes administration, although full tumor regression was only observed in 20% of mice, similar to the previous observed for empty PD-L1 targeted liposomes but at a dose four times higher (100 µg/mouse) [30]. In addition, in this work the survival rate as well as the life span increased significantly, the latter by 1 month, suggesting that another cycle of treatment might maintain the control of tumor progression or even increase the number of cured mice. Note that although all treatments were able to trigger an initial partial response at the end of treatments, it seemed insufficient to overcome the tumor growth. Hence, only LPF were able to achieve an antitumor immune activation able to control the tumor growth in a very aggressive melanoma mice model.

## Conclusion

The PD-L1 targeted Dox liposomal formulation here developed and assayed in a melanoma murine model, has demonstrated an enhanced contribution of both components, α-PD-L1 and Dox, in the control of tumor growth and elimination, enabling host antitumor immune modulation. To date this is the first successful chemo-immunotherapeutic approach combined in an immunoliposome.

Furthermore, this formulation represents a versatile nano-platform for the encapsulation of different therapeutic molecules or co-encapsulation with fluorescent probes for theragnostic application not only in cancer, but even, in other illnesses.

## Materials and methods

### Materials

The lipids Hydrogenated Soy l-α-phosphatidylcholine (HSPC), Cholesterol (CH), 1,2-Distearoyl-sn-Glycero-3-Phosphoethanolamine-*N*-[Methoxy(Polyethylene Glycol)-2000] (DSPE-PEG_2000_) and 1,2-Distearoyl-sn-Glycero-3-Phosphoethanolamine-*N*-[Maleimide(Polyethylene Glycol2000)] (DSPE-PEG_2000_-Mal) were purchased from Avanti polar lipids Inc. (Alabama, USA). PD-10 desalting prepacked columns containing Sephadex G-25 resin were purchased from GE Healthcare Life Sciences (Pittsburgh,USA). Chloroform, Methanol, Trypsin–EDTA, β-mercaptoethanol, Hepes, Sodium Chloride, β-Mercapto­ethylamine hydrochloride (MEA), EDTA, Hepes, phorbolmyristate acetate(PMA), Ionomycin, Daunorubicin and Triton X-100 were purchased from Sigma Aldrich (Madrid, Spain). Dox was purchased from the Pharmacy Service (Clínica Universidad de Navarra, Pamplona, Spain). The cell culture medium Roswell Park Memorial Institute medium (RPMI-Glutamax), Penicillin–Streptomycin (P/S) and Fetal Bovine Serum (FBS) were obtained from GIBCO (Madrid, Spain). α-PD-L1 (Clone 10F.9G2) was obtained from BioXCell (West Lebanon, USA). Collagenase and DNAse were purchased from Roche® (Basel, Switzerland) and ACK lysis buffer was obtained from Invitrogen (California, USA). Tris (2-carboxyethyl) phosphine (TCEP) and immobilized pepsin were obtained from Thermofisher (Massachusetts,USA). Centrifugal Filter Units (Amicons) of 10,000 and 30,000 MWCO were obtained from Merck (Darmstadt, Germany). The Recombinant Mouse B7-H1/Fc chimera was obtained from R&D Systems (Minnesota, USA). iTAgTM MHC Class I Murine Tetramer-SA-PE was obtained from Immudex (Copenhagen, Denmark) and (FITC)-conjugated α-mouse CD8α, Zombie-APCCy7 and APC α-mouse CD279 (PD-1) were purchased from BioLegend (California, USA). GolgiStop™ and GolgiPlug™ were obtained from BD Biosciences (California, USA).

### Methods

#### Targeted liposome preparation

##### Development of Dox liposomes

Liposomes were prepared by the film-hydration method combined with a pH gradient for active drug loading [30, 38, 52].

Briefly, HSPC:CH:DSPE-PEG_2000_ at a molar ratio of 1.85:1:0.12, were dissolved in a solution of chloroform:methanol [9:1 (v/v)]. Organic solvents were removed by rotary evaporation (Büchi Waterbath B-480, Switzerland) at 65 °C obtaining the film, which was hydrated with ammonium sulphate (250 mM) at pH 5.5 under constant stirring. The liposomal solution was extruded five times through a polycarbonate membrane with a drain disk. To obtain a homogeneous population of liposomes, membranes with different pore size were used, 200 nm, 100 nm and 80 nm.

Ammonium sulphate buffer was exchanged with Hepes saline buffer (Hepes 10 mM, EDTA 5 mM and NaCl 150 mM; pH 6.7) through a PD-10 column and LPs were incubated with Dox at a molar ratio of 0.1:1(Dox:Lipid) in a thermoshaker (Vortemp 56, Labnet, USA) for 1 h at 60 °C. Non-encapsulated Dox was removed by ultracentrifugation at 40,000 rpm for 2 h at 4 °C and the final Dox liposomal formulation was kept in Hepes saline at 4 °C until use.

##### α-PD-L1 Fab’ fragments

The α-PD-L1 monovalent variable fragment (Fab’) was obtained according to the methodology reported by Merino et al. [30]. Briefly, in the first step, α-PD-L1 was digested with in mobilized pepsin at 37 °C for 3 h in sodium acetate. This solution was washed with Hepes saline buffer using the Amicon system (MWCO 30 K) to collect F(ab’)_2_ fragments, which were incubated at 37 °C for 2 h with MEA solution (15 mM) and filtered (Amicon system, MWCO 10 K) to expose out sulfhydryl groups. These fragments were able to react with DSPE-PEG_2000_-Mal to form thioether bonds. To prevent the disulfide crosslink in the fragments, these were treated for 1 h at 37 °C with TCEP (500 mM). The mixture was washed again, collecting the Fab’ fragments at 4 °C that were used within the next 4 h.

### Preparation of Dox immunoliposomes

**Fig. 10:**
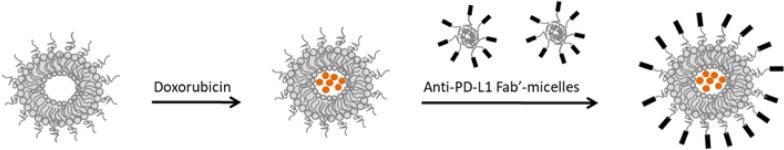
Schematic representation of the different steps used for Targeted Doxorubicin liposomes formulation

The post insertion method [53, 54], based on the preparation of specific targeted micelles, was used to formulate α-PD-L1-Fab’ Dox liposomes. Micelles, composed of DSPE-PEG_2000_-Mal, were prepared at pH 6.7 in Hepes saline buffer and incubated overnight (ON) at 4 °C with Fab’ fragments, at a molar ratio of 750:1 (lipid:ligand) under constant stirring. Targeted micelles and preformed LPD were incubated together for 1 h at 60 °C, as is shown in Fig. 10. Formulated immunoliposomes were incubated with 1 mM of l-cysteine to quench free maleimide groups. In order to prevent possible Dox release during this procedure, targeted liposomes were ultracentrifuged at 28,000 rpm, 4 °C for 3 h and resuspended in Hepes saline at 4 °C until use.

### Characterization of liposomes

#### Physicochemical characterization

The different Dox formulations were characterized in terms of particle size, polydispersity index (PDI) and Zeta potential by laser diffractometry using a Zetasizer Nano Series system (Malvern Instruments, UK).

The encapsulation efficiency of Dox was measured by spectrophotometry (λ 485 nm), and loading efficiency was calculated by the following formula:

$$EE\left(\%\right)=\frac{Fa}{Ia}*100$$

where, *EE* represents the percentage of drug entrapment efficiency; *Fa,* the amount of drug encapsulated in liposomes divided by the final amount of lipids and *Ia,* the initial amount of the drug divided by the initial amount of lipids.

The lipid concentration in each batch was measured by the phosphate assay [55], whereas the ligand conjugation efficiency, was quantified using the Coomassie Protein assay reagent (Bio-Rad, California, USA). The number of attached ligand molecules was calculated according to the amount of protein and lipid, using the Avogadro’s number [56]. Long-term stability at 4 °C was measured over 3 months, characterizing particle size, PDI and Dox encapsulation at certain time points.

#### Non-targeted and targeted liposomes stability

Dox release was measured by fluorimetry (λ_ex_ 485 nm and λ_em_ 595 nm) using aliquots of 5 mM lipids (LPD y LPF) placed in 100% FBS and incubated at 37 °C. The Dox signal was recorded every second for 1 h. Afterwards, aliquots were treated with TritonX-100 (1%) to measure the encapsulated Dox. The accumulative release curves were built using the following formula:

$$\% R = \left( {\frac{Qa}{{Qt}}} \right) \times 100$$

where, *Qa* represents the amount of drug measured in the collected samples, and *Qt*, the total encapsulated amount. This experiment was evaluated in duplicate in three independent formulations.

### In vitro studies

B16OVA murine melanoma cell line was kindly provided by Dr. Sandra Hervás-Stubbs (CIMA, Pamplona, Spain). The cell line is derived from B16-F10 and transfected with chicken ovalbumin, a protein that acts as a surrogate tumor antigen in the presence of Geneticin (50 mg/mL, Lonza®, Spain). B16OVA cells over-expressing PD-L1 [30] were maintained in a mixture of RPMI-Glutamax cell culture medium, 10% FBS, 1% P/S, 2% Hepes and 50 mM of β-mercaptoethanol under standard conditions. The presence of Mycoplasma was regularly tested (Lonza, Spain).

### Cellular interaction of targeted and non-targeted liposomes

B16OVA cells were seeded at a density of 8 × 10^4^ cells/well in 12-well plates. Twenty-four hours later cells were exposed to 5 µg/mL of Dox encapsulated in LPD or LPF for 4 and 24 h at 37 °C. Afterwards, cells were washed and detached in 200 µL PBS. Dox-specific fluorescence was measured on a BD FACS Calibur™ flow cytometer (BD biosciences) and analyzed using FlowJo software v 7.6.1 (TreeStar, USA).

Additionally, images were also captured by confocal microscopy. Cells were seeded in culture slides chambers (BD Falcon, Bedford, USA) at a density of 3 × 10^4^ cells/well and treated with 5 µg/mL of Dox according to the protocol described above. After treatments, cells were washed several times and the nuclei stained in green with Hoechst (1:1000 in PBS) during 10 min at 37 °C. Images were acquired at 40× of magnification using the Axio Cam MR3 video camera connected to the Zeiss Imager M1 microscope (Carl Zeiss AG, Oberkochen, Germany) equipped with epifluorescence optics and Axiovison software (4.6.3.0 version).

### Cytotoxicity evaluation in PD-L1^+^ cells

B16OVA cells were seeded in 96 wells plates (flat bottom) at a density of 5 × 10^3^ cells/well. Twenty-four hours later, cells were exposed for 4 h to different Dox concentrations (from 0.001 to 100 µM), free and encapsulated in LPD or LPF. Wells, washed three times with PBS, were incubated at 37 °C in fresh medium for 72 h. Cytotoxicity was tested using the sulphorodamine B assay [57] and data, expressed as IC_50_ (Dox concentration able to inhibit the 50% of cell proliferation in comparison to control or non-treated cells) were determined by a non-linear regression plotting cell survival vs. log concentration (GraphPad Prism software v 6.01).

### In vivo evaluation of liposomal formulations

**Fig. 11:**
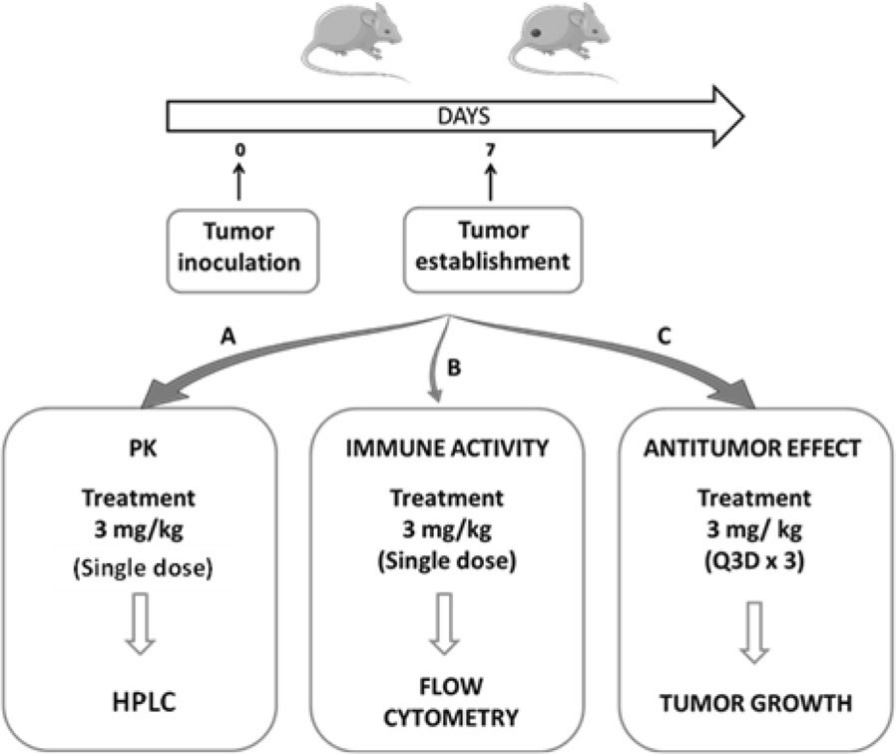
Schematic representation of the three in-vivo studies performed in this work

Female C57BL/6 mice (20 g or 5 weeks, supplied by Harlan, Barcelona, Spain) were housed in plastic cages under standard and sterile conditions (25 °C, 50% relative humidity, 12 h dark/light) with water and food ad libitum. All experiments were performed according to European animal care regulations, ARRIVE guidelines and the protocol approved by the Ethics Committee of the University of Navarra (Ref. 046-14). Mice were subcutaneously injected in the right flank with B16OVA melanoma cells in 100 μl of PBS (5 × 10^5^ cells/mouse). Tumor growth and body weight were monitored twice a week. When tumor size reached approximately a mean diameter of 5–6 mm, mice were randomly divided into several groups to assay different experiments (Fig. 11).

### Pharmacokinetics behaviour of the different Dox-formulations

C57BL/6J female mice (n = 27) inoculated with B16OVA cells were divided into three groups: free Dox, LPD and LPF, receiving a single intravenously dose of Dox, 3 mg/kg. Blood samples were collected in EDTA tubes (SARSTEDT, Germany) via the maxillofacial vein at different time points post-injection, from 5 min to 168 h. Plasma was obtained by blood centrifugation at 3500 rpm for 15 min and used for Dox quantification by HPLC. The analytical system (HP 1100 Series Agilent) was equipped with a fluorescence detector (G1321A FLD) and Agilent ChemStation software (Germany). The chromatographic separation was performed with a Gemini® C_18_ 110 Å column (150 × 3 mm, particle size 5 µm, Phenomenex®). The mobile phase consisted of water (pH 2.5, acidified with orthophosphoric acid)/acetonitrile and the flow rate was 0.7 mL/min under gradient conditions, starting at 80/20 to change at 70/30 during the next 3 min, 0/100 in the next 9 min and achieving the initial condition at 10 min. The detection was performed at λ_em_ 480 nm and λ_ex_ 558 nm. Calibration curves were prepared in 100 µL of murine plasma (Plasma Balb/C, GeneTex, Irvine, USA) using a stock solution of Dox and the internal standard, Daunorubicin (15 µg/mL). The linear range was from 0.0125 to 1.5 µg/mL.

Time profiles of Dox plasma concentration administered under different formulations were built using the average value corresponding to the three mice used at each time point. The area under the plasma concentration versus time curve (AUC_0-∞_) was calculated by the linear trapezoidal method. For the extrapolated area, AUC_last_obs-∞_, the last concentration (C_last_obs_) was divided by the slope (*k*) obtained from the terminal portion of the curve using data log-transformed:

$${[AUC]}_{last\_obs}^{\infty }={C}_{last\_obs}/k$$

Other parameters such as elimination half-life (t_1/2_), plasma clearance (*CL*) and volume of distribution (*V*_*d*_) were estimated according to the equations: ${t}_{1/2}=0.693/k$, $CL=Dose/{AUC}_{0-\infty }$ and $Vd=CL/k$, respectively.

### Characterization of the immunological effect induced by liposomal formulations

The immune system activation provided by the different formulations was studied in B16OVA tumor-bearing mice. Briefly, when tumors reached the optimal size, mice were randomly divided into different groups: Control, free Dox, LPD, LPD plus free α-PD-L1 (1.2 mg/kg) and LPF. Treatments consisted of a single i.v. dose of 3 mg/kg of Dox. One week later, mice were sacrificed and tumors, spleens and lymph nodes were collected for further analysis.

Tumor tissues were digested with 5 mL collagenase/DNase (10:1) at 37 °C for 30 min, neutralized with 50 µL of EDTA and centrifuged for 2 min at 1800 rpm. Pellets were lysed with 1 mL of ACK lysis buffer (Lonza, USA), neutralized with PBS and transferred to 96 well microtiter plates for staining.

After 10 min incubation at room temperature with Zombie-APC-Cy7 (1:1000 v/v in PBS), cells were again incubated 10 min at 4 °C with FC blocking antibody (1:200, v/v) in Facs buffer (5% FBS and 0.5% EDTA in PBS) to reduce unspecific bindings. Samples were incubated with iTAgTM MHC Class I Murine Tetramer-SA-PE (1:50) for 30 min at 4 °C, washed and treated for 15 min at 4 °C with a mixture of (FITC)-conjugated α-mouse CD8α (1:200) and APC α-mouse CD279 (PD-1) (1:200). Cells were washed and resuspended in Facs buffer at 4 °C until flow cytometry analysis.

Spleen samples were analyzed by the Elispot assay. The day before the experiment, a 96 well Elispot microtiter plate was coated with the capture antibody overnight (1:200 dilution in PBS) at 4 °C. Twenty-four hours later, the plate was washed and blocked for 2 h by adding complete RPMI culture medium. Samples were processed in the lysis buffer for 2 min, neutralized with RPMI culture medium and centrifuged at 1800 rpm for 2 min. The pellets, resuspended in complete RPMI medium, were incubated overnight at a density of 1 × 10^6^ cells/well with SIINFEKL peptide (1 µg/mL) or ovalbumin protein (10 µg/mL) at 37 °C. Twenty four hours later, cells were washed and incubated for 2 h with the detection biotinylated antibody (1:250) prepared in PBS and 10% FBS. These cells were incubated for 1 h with Streptavidin-HRP (1:100) and treated in darkness with a substrate solution (AEC substrate set, BD) until the spots emerged. The reaction was stopped by adding water and the spots were counted with the ImmunoSpot program.

Lymph nodes were mashed in PBS and stimulated for 5 h with PMA (50 µg/mL) and Ionomycin (1 µg/mL). For intracellular staining, cells were incubated with BD GolgiStop™ and GolgiPlug™ (BD, 1:1000 dilutions), stained with PE labelled H-2 kb/OVA257-264-tetramers (MBL) and also with fluorochrome-conjugated antibodies against CD8, NKp46 and CD44, in the presence of purified α-CD16/32. In the next step, cells were fixed and permeabilized with the BD Fixation/Perm buffer (BD) to stain with granzyme B (GB11) antibody. Samples were acquired on a FACS Canto-II cytometer (BD Biosciences) and data were analyzed using FlowJo software (TreeStar). Cell death was detected with Zombie NIR Fixable dye (1:1000).

### Antitumor efficacy study

**Fig. 12:**
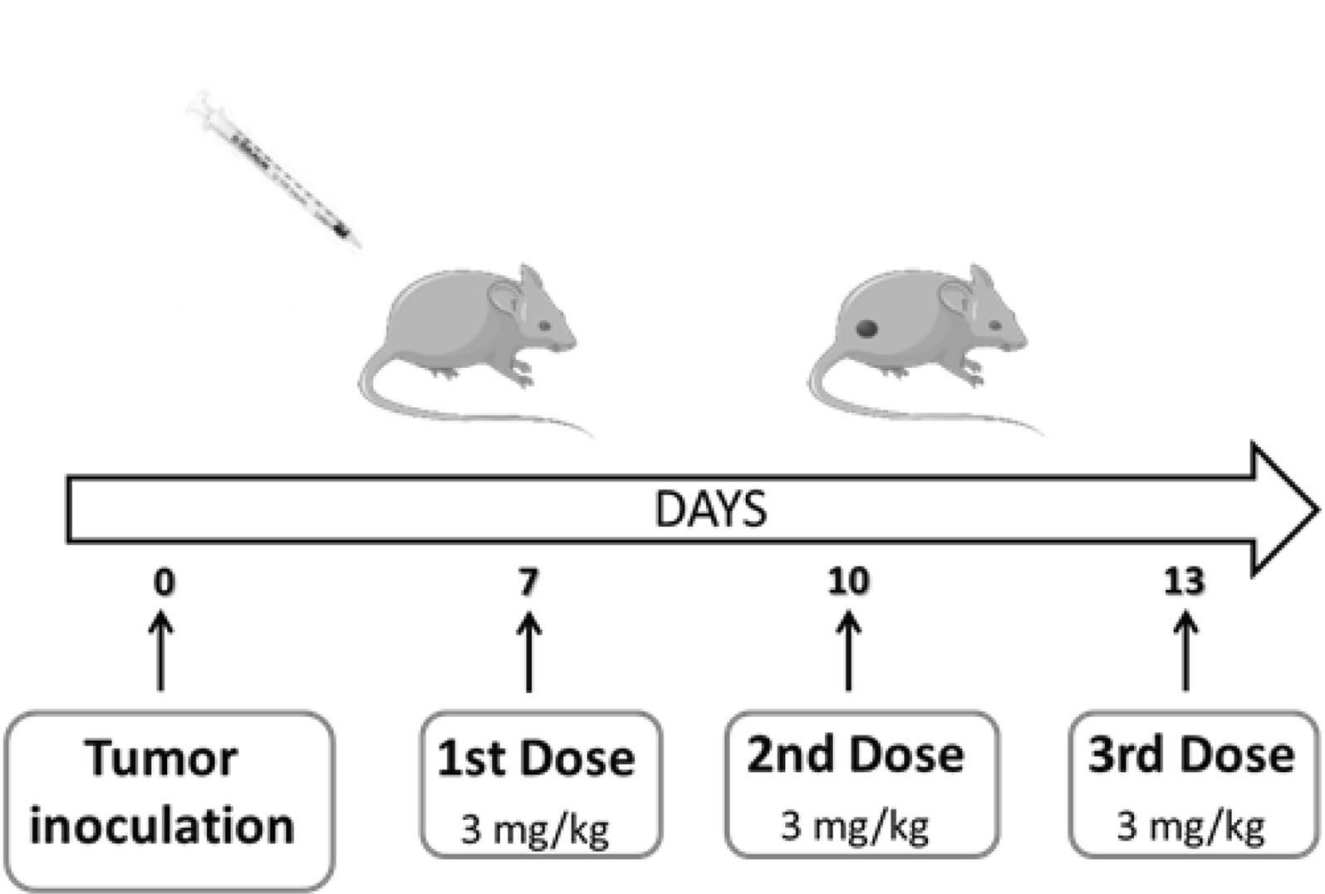
Schematic representation of the dose schedule applied to evaluate the antitumor response in the in-vivo study

Antitumor efficacy was studied in C57BL/6 J tumor-bearing mice (n = 60) randomly grouped into control, free Dox, LPD, LPD/α-PD-L1 and LPF (Fig. 12). Treatments consisted of 3 mg/kg Dox administered intravenously every 72 h, for three doses, and for α-PD-L1, in the combination, was 28 µg/mouse, the dose equivalent to the conjugated to LPF. Mice body weight and other side effects were measured over time until the end of the experiment. Tumor size was calculated by the following formula:

$$V=\frac{\left({a}^{2}*b\right)}{2}$$

where, a represents the shortest and b, the largest dimensions of the tumor.

### Statistical analysis

The statistical analysis was performed using GraphPad Prism 5 software (version 6.01) (GraphPad Software, San Diego, USA). The one-way ANOVA test was used to compare all groups together, followed by the Bonferroni’s test for checking the differences between groups. Statistical analysis of survival data was performed in RStudio (version 3.6.3) using the Survfit function from the survival package that computes the Kaplan–Meier estimator for truncated and/or censored data. The log-rank test was used to compare the different treatment groups. Statistical significance was set at 0.05.

## Supplementary Information

**Additional file 1.** Supplementary results.

## Data Availability

The datasets used and/or analyzed during the current study are available from the corresponding author on reasonable request.

## Abbreviations

AUC: Area under the curve of Dox plasma concentrations vs. time
Cl: Total plasma Clearance
C_last obs_: The last Dox concentration observed
Fab’: Monovalent-variable fragments of an antibody
Dox: Doxorubicin
EE: Encapsulation efficiency
ICD: Immunogenic tumor cell death
ICI: IC inhibitors
k: Slope representing the elimination rate constant
LP: Empty liposomes
LPD: Doxorubicin liposomes
LPF: Targeted doxorubicin liposomes
MIF: Mean fluorescent signal
ON: Overnight
PDI: Polydispersity index
PD-1: Programmed Death-1
PD-L1: Programmed Death Ligand-1
RECIST: Response Evaluation Criteria In Solid Tumors
(t_1/2_): Elimination Half-life
Vd: Apparent Volume of distribution

## References

[1] Mittal D, Gubin MM, Schreiber RD, Smyth MJ (2014). New insights into cancer immunoediting and its three component phases-elimination, equilibrium and escape. Curr Opin Immunol.

[2] Shi T, Ma Y, Yu L, Jiang J, Shen S, Hou Y, Wang T (2018). Cancer immunotherapy: a focus on the regulation of immune checkpoints. Int J Mol Sci.

[3] Merelli B, Massi D, Cattaneo L, Mandalà M (2014). Targeting the PD1/PD-L1 axis in melanoma: biological rationale, clinical challenges and opportunities. Crit Rev Oncol Hematol.

[4] Philips GK, Atkins M (2015). Therapeutic uses of anti-PD-1 and anti-PD-L1 antibodies. Int Immunol.

[5] Blank CU (2014). The perspective of immunotherapy: new molecules and new mechanisms of action in immune modulation. Curr Opin Oncol.

[6] Topalian SL, Drake CG, Pardoll DM (2012). Targeting the PD-1/B7-H1(PD-L1) pathway to activate anti-tumor immunity. Curr Opin Immunol.

[7] Robert C, Soria J-C, Eggermont AMM (2013). Drug of the year: programmed death-1 receptor/programmed death-1 ligand-1 receptor monoclonal antibodies. Eur J Cancer.

[8] Contreras-Sandoval AM, Merino M, Vasquez M, Trocóniz IF, Berraondo P, Garrido MJ (2016). Correlation between anti-PD-L1 tumor concentrations and tumor-specific and nonspecific biomarkers in a melanoma mouse model. Oncotarget.

[9] Brahmer JR, Tykodi SS, Chow LQMM, Hwu W-JJ, Topalian SL, Hwu P, Drake CG, Camacho LH, Kauh J, Odunsi K, Pitot HC, Hamid O, Bhatia S, Martins R, Eaton K, Chen S, Salay TM, Alaparthy S, Grosso JF, Korman AJ, Parker SM, Agrawal S, Goldberg SM, Pardoll DM, Gupta A, Wigginton JM (2012). Safety and activity of anti-PD-L1 antibody in patients with advanced cancer. N Engl J Med.

[10] Borch TH, Donia M, Andersen MH, Svane IM (2015). Reorienting the immune system in the treatment of cancer by using anti-PD-1 and anti-PD-L1 antibodies. Drug Discov Today.

[11] Inthagard J, Edwards J, Roseweir AK (2019). Immunotherapy: enhancing the efficacy of this promising therapeutic in multiple cancers. Clin Sci.

[12] Thallinger C, Füreder T, Preusser M, Heller G, Müllauer L, Höller C, Prosch H, Frank N, Swierzewski R, Berger W, Jäger U, Zielinski C (2018). Review of cancer treatment with immune checkpoint inhibitors: current concepts, expectations, limitations and pitfalls. Wien Klin Wochenschr.

[13] Emens LA (2010). Chemoimmunotherapy. Cancer J.

[14] Opzoomer JW, Sosnowska D, Anstee JE, Spicer JF, Arnold JN (2019). Cytotoxic chemotherapy as an immune stimulus: a molecular perspective on turning up the immunological heat on cancer. Front Immunol.

[15] Tesniere A, Apetoh L, Ghiringhelli F, Joza N, Panaretakis T, Kepp O, Schlemmer F, Zitvogel L, Kroemer G (2008). Immunogenic cancer cell death: a key-lock paradigm. Curr Opin Immunol.

[16] Locher C, Conforti R, Aymeric L, Ma Y, Yamazaki T, Rusakiewicz S, Tesnière A, Ghiringhelli F, Apetoh L, Morel Y, Girard J-P, Kroemer G, Zitvogel L (2010). Desirable cell death during anticancer chemotherapy. Ann N Y Acad Sci.

[17] Kroemer G, Galluzzi L, Kepp O, Zitvogel L (2013). Immunogenic cell death in cancer therapy. Annu Rev Immunol.

[18] Tila D, Ghasemi S, Yazdani-Arazi SN, Ghanbarzadeh S (2015). Functional liposomes in the cancer-targeted drug delivery. J Biomater Appl.

[19] Tacar O, Sriamornsak P, Dass CR (2013). Doxorubicin: an update on anticancer molecular action, toxicity and novel drug delivery systems. J Pharm Pharmacol.

[20] Barenholz Y (2012). Doxil®–the first FDA-approved nano-drug: lessons learned. J Control Release.

[21] Maeda H (2015). Toward a full understanding of the EPR effect in primary and metastatic tumors as well as issues related to its heterogeneity. Adv Drug Deliv Rev.

[22] Maeda H (2001). The enhanced permeability and retention (EPR) effect in tumor vasculature: the key role of tumor-selective macromolecular drug targeting. Adv Enzyme Regul.

[23] Maeda H, Wu J, Sawa T, Matsumura Y, Hori K (2000). Tumor vascular permeability and the EPR effect in macromolecular therapeutics: a review. J Control Release.

[24] Merino M, Zalba S, Garrido MJ (2018). Immunoliposomes in clinical oncology: state of the art and future perspectives. J Control Release.

[25] Eloy JO, Petrilli R, Trevizan LNF, Chorilli M (2017). Immunoliposomes: a review on functionalization strategies and targets for drug delivery. Colloids Surf B Biointerfaces.

[26] Wang D, Sun Y, Liu Y, Meng F, Lee RJ (2018). Clinical translation of immunoliposomes for cancer therapy: recent perspectives. Expert Opin Drug Deliv.

[27] Bertucci F, Finetti P, Perrot D, Leroux A, Collin F, Le Cesne A, Coindre JM, Blay JY, Birnbaum D, Mamessier E (2017). PDL1 expression is a poor-prognosis factor in soft-tissue sarcomas. Oncoimmunology.

[28] Tamura T, Ohira M, Tanaka H, Muguruma K, Toyokawa T, Kubo N, Sakurai K, Amano R, Kimura K, Shibutani M, Maeda K, Hirakawa K (2015). Programmed death-1 ligand-1 (PDL1) expression is associated with the prognosis of patients with stage II/III gastric cancer. Anticancer Res.

[29] Chen XY, Zhang J, Hou LD, Zhang R, Chen W, Fan HN, Huang YX, Liu H, Zhu JS (2018). Upregulation of PD-L1 predicts poor prognosis and is associated with miR-191-5p dysregulation in colon adenocarcinoma. Int J Immunopathol Pharmacol.

[30] Merino M, Contreras A, Casares N, Troconiz IF, ten Hagen TL, Berraondo P, Zalba S, Garrido MJ (2019). A new immune-nanoplatform for promoting adaptive antitumor immune response. Nanomed Nanotechnol Biol Med.

[31] Hoos A, Eggermont AMM, Janetzki S, Hodi FS, Ibrahim R, Anderson A, Humphrey R, Blumenstein B, Old L, Wolchok J (2010). Improved endpoints for cancer immunotherapy trials. J Natl Cancer Inst.

[32] Vrankar M, Unk M (2018). Immune RECIST criteria and symptomatic pseudoprogression in non-small cell lung cancer patients treated with immunotherapy. Radiol Oncol.

[33] Ascierto PA, Agarwala SS, Eggermont A, Gershenwald JE, Grob JJ, Hamid O, Michielin O, Postow M, Puzanov I, Zarour HM, Caracò C, Testori A (2020). The Great Debate at “melanoma Bridge”, Naples, December 7th 2019. J Transl Med..

[34] Sau S, Petrovici A, Alsaab HO, Bhise K, Iyer AK (2019). PDL-1 antibody drug conjugate for selective Chemo-guided immune modulation of cancer. Cancers..

[35] Lee P, Gujar S (2018). Potentiating prostate cancer immunotherapy with oncolytic viruses. Nat Rev Urol.

[36] Liu Y, Chen XG, Yang PP, Qiao ZY, Wang H (2019). Tumor Microenvironmental pH and enzyme dual responsive polymer-liposomes for synergistic treatment of cancer immuno-chemotherapy. Biomacromol.

[37] Wei L, Yu F, Meng Y (2020). Preparation of programmed cell death-ligand 1 antibody nanoparticles based on nude mouse model and its therapeutic effect on lung cancer. J Nanosci Nanotechnol.

[38] Saeed M, Zalba S, Seynhaeve ALB, Debets R, Ten Hagen TLM (2019). Liposomes targeted to MHC-restricted antigen improve drug delivery and antimelanoma response. Int J Nanomed.

[39] Mamot C, Ritschard R, Wicki A, Küng W, Schuller J, Herrmann R, Rochlitz C (2012). Immunoliposomal delivery of doxorubicin can overcome multidrug resistance mechanisms in EGFR-overexpressing tumor cells. J Drug Target.

[40] Kaminskas LM, McLeod VM, Kelly BD, Sberna G, Boyd BJ, Williamson M, Owen DJ, Porter CJH (2012). A comparison of changes to doxorubicin pharmacokinetics, antitumor activity, and toxicity mediated by PEGylated dendrimer and PEGylated liposome drug delivery systems. Nanomed Nanotechnol Biol Med.

[41] Gabizon AA, Barenholz Y, Bialer M (1993). Prolongation of the circulation time of doxorubicin encapsulated in liposomes containing a polyethylene glycol-derivatized phospholipid: pharmacokinetic studies in rodents and dogs. Pharm Res An Off J Am Assoc Pharm Sci..

[42] Sun JY, Zhang D, Wu S, Xu M, Zhou X, Lu XJ, Ji J (2020). Resistance to PD-1/PD-L1 blockade cancer immunotherapy: mechanisms, predictive factors, and future perspectives. Biomark Res.

[43] Wang J, Wu Z, Pan G, Ni J, Xie F, Jiang B, Wei L, Gao J, Zhou W (2018). Enhanced doxorubicin delivery to hepatocellular carcinoma cells via CD147 antibody-conjugated immunoliposomes. Nanomed Nanotechn Biol Med.

[44] Lucas AT, Herity LB, Kornblum ZA, Madden AJ, Gabizon A, Kabanov AV, Ajamie RT, Bender DM, Kulanthaivel P, Sanchez-Felix MV, Havel HA, Zamboni WC (2017). Pharmacokinetic and screening studies of the interaction between mononuclear phagocyte system and nanoparticle formulations and colloid forming drugs. Int J Pharm.

[45] Lee MS, Dees EC, Wang AZ (2017). Nanoparticle-delivered chemotherapy: old drugs in new packages. Oncology..

[46] Chen G, Huang AC, Zhang W, Zhang G, Wu M, Xu W, Yu Z, Yang J, Wang B, Sun H, Xia H, Man Q, Zhong W, Antelo LF, Wu B, Xiong X, Liu X, Guan L, Li T, Liu S, Yang R, Lu YY, Dong L, McGettigan S, Somasundaram R, Radhakrishnan R, Mills G, Lu YY, Kim J, Chen YH, Dong H, Zhao Y, Karakousis GC, Mitchell TC, Schuchter LM, Herlyn M, Wherry EJ, Xu X, Guo W (2018). Exosomal PD-L1 contributes to immunosuppression and is associated with anti-PD-1 response. Nature.

[47] Berghoff AS, Pajenda S, Ilhan-Mutlu A, Widhalm G, Dieckmann K, Hainfellner JA, Wagner L, Zielinski C, Birner P, Bartsch R, Preusser M (2015). Plasma PD-L1 concentration in patients with brain metastases from solid tumors. J Clin Oncol.

[48] Sponaas AM, Moharrami NN, Feyzi E, Standal T, Rustad EH, Waage A, Sundan A (2015). PDL1 expression on plasma and dendritic cells in myeloma bone marrow suggests benefit of targeted anti PD1-PDL1 therapy. PLoS ONE.

[49] Xiong H, Mittman S, Rodriguez R, Moskalenko M, Pacheco-Sanchez P, Yang Y, Nickles D, Cubas R (2019). Anti-PD-L1 treatment results in functional remodeling of the macrophage compartment. Cancer Res.

[50] Gurung S, Khan F, Gunassekaran GR, Do Yoo J, Poongkavithai Vadevoo SM, Permpoon U, Kim SH, Kim HJ, Kim IS, Han H, Park JH, Kim S, Lee B (2020). Phage display-identified PD-L1-binding peptides reinvigorate T-cell activity and inhibit tumor progression. Biomaterials..

[51] Chen DS, Mellman I (2013). Oncology meets immunology: the cancer-immunity cycle. Immunity.

[52] Zalba S, Seynhaeve ALB, Brouwers JF, Süss R, Verheij M, Ten Hagen TLM (2020). Sensitization of drug resistant sarcoma tumors by membrane modulation: via short chain sphingolipid-containing nanoparticles. Nanoscale.

[53] Ishida T, Iden DL, Allen TM (1999). A combinatorial approach to producing sterically stabilized (Stealth) immunoliposomal drugs. FEBS Lett.

[54] Rothdiener M, Beuttler J, Messerschmidt SKE, Kontermann RE (2010). Antibody targeting of nanoparticles to tumor-specific receptors: immunoliposomes. Methods Mol Biol.

[55] Rouser G, Fkeischer S, Yamamoto A (1970). Two dimensional then layer chromatographic separation of polar lipids and determination of phospholipids by phosphorus analysis of spots. Lipids.

[56] Saeed M, van Brakel M, Zalba S, Schooten E, Rens JAPP, Koning GA, Debets R, ten Hagen TLMM (2016). Targeting melanoma with immunoliposomes coupled to anti-MAGEAI TCR-like single-chain antibody. Int J Nanomedicine.

[57] Vichai V, Kirtikara K (2006). Sulforhodamine B colorimetric assay for cytotoxicity screening. Nat Protoc.

