# Supplementary material for: Dual activity of PD-L1 targeted Doxorubicin immunoliposomes promoted an enhanced efficacy of the antitumor immune response in melanoma murine model
Source: J Nanobiotechnology. 2021 Apr 13;19:102. doi: 10.1186/s12951-021-00846-z (PMC8042980; doi:10.1186/s12951-021-00846-z)
Supplement: Supplementary file 1 — Additional file 1. Supplementary results. [file 12951_2021_846_MOESM1_ESM.docx]

**SUPPLEMENTARY MATERIAL**

*Table S1. Lipid composition of the different formulations developed and assayed in this work. Encapsulation Efficiency (EE) of Dox was higher than 90% whereas the efficiency of PD-L1 Fab´ fragments conjugation was about 80-90 molecules per liposome.*

| **Formulation ID** | **Lipid composition** | **Molar ratio** | **Dox**  **EE(%)** | **PD-L1 Fab´** |
| --- | --- | --- | --- | --- |
| **LP** | HSPC:CH:DSPE-PEG_2000_ | 1.85:1:0.12 | - | - |
| **LPD** | HSPC:CH:DSPE-PEG_2001_ | 1.85:1:0.12 | > 90% | - |
| **LPF** | HSPC:CH:DSPE-PEG_2002_ | 1.85:1:0.12 | > 90% | 80-90 molecules/liposome |

*Dox: doxorubicin; LP: Empty conventional liposome; LPD: Conventional Dox liposomes; LPF: Targeted Dox liposomes.*

C)

B)

A)

*Figure S1. HPLC analysis of Dox after plasma extraction. The first pick correspond to Dox signal and the second to Daunorubicin standard. A) Free Dox after 5 minutes of administration; B) LPD after 4 hours of administration; C) LPF after 4 hours of administration.*

*Dox: doxorubicin; LPD: conventional Doxorubicin liposomes; LPF: targeted doxorubicin liposomes*

*Figure S2. Mice weight evolution of the different treatment groups. Mice were administered with B16OVA cells subcutaneously. Seven days later mice were divided into different groups: Control, free Dox, LPD, LPD + 28 µG of free α-PD-L1 and LPF. Three doses of 3 mg/kg of Dox were administered every three days. Data correspond to the average of individual weight changes of each treatment group. (n=12)*

*Dox: doxorubicin; LPD: conventional Doxorubicin liposomes; LPF: targeted doxorubicin liposomes*

*Figure S3. Ex vivo liposome interaction assay. Bone marrow cells were differenciated to myeloid populations prior to be exposed to targeted and non-targeted fluorescent liposomes for 4 h. A) Geomean fluorescent signal of myeloid cells after be exposed to different lipid amounts of targeted and non-targeted liposomes; B) PD-L1 expression of control and treated cells with different lipid amounts of targeted and non-targeted liposomes.*

*LP: conventional empty liposomes; LP Fab: targeted empty liposomes*
